# UV-Radiation: From Physics to Impacts

**DOI:** 10.3390/ijerph14020200

**Published:** 2017-02-17

**Authors:** Hanns Moshammer, Stana Simic, Daniela Haluza

**Affiliations:** 1 Department for Environmental Health, Center for Public Health, Medical University of Vienna, Vienna 1090, Austria; daniela.haluza@meduniwien.ac.at; 2 Institute for Meteorology, University of Natural Resources and Life Sciences, Vienna 1180, Austria; stana.simic@boku.ac.at

**Keywords:** ultraviolent radiation, health counseling, adverse and beneficial effects

## Abstract

Ultraviolet (UV) radiation has affected life at least since the first life forms moved out of the seas and crawled onto the land. Therefore, one might assume that evolution has adapted to natural UV radiation. However, evolution is mostly concerned with the propagation of the genetic code, not with a long, happy, and fulfilling life. Because rickets is bad for a woman giving birth, the beneficial effects of UV-radiation outweigh the adverse effects like aged skin and skin tumors of various grades of malignancy that usually only afflict us at older age. Anthropogenic damage to the stratospheric ozone layer and frighteningly high rates of melanoma skin cancer in the light-skinned descendants of British settlers in Australia piqued interest in the health impacts of UV radiation. A changing cultural perception of the beauty of tanned versus light skin and commercial interests in selling UV-emitting devices such as tanning booths caught public health experts off-guard. Counseling and health communication are extremely difficult when dealing with a “natural” risk factor, especially when this risk factor cannot (and should not) be completely avoided. How much is too much for whom or for which skin type? How even measure “much”? Is it the (cumulative) dose or the dose rate that matters most? Or should we even construct a more complex metric such as the cumulative dose above a certain dose rate threshold? We find there are still many open questions, and we are glad that this special issue offered us the opportunity to present many interesting aspects of this important topic.

## 1. Introduction

We were thrilled when the journal invited us to serve as guest editors for a special issue about ultraviolet (UV) radiation. We are three Austrian scientists: Stana Simic (SS) as a meteorologist has long-standing experience in UV research as a coordinator of the Austrian UV monitoring network [1,2] and as an operator of the UV spectrometer at Hoher Sonnblick, an Alpine observatory in 3106 m [3,4]. Daniela Haluza (DH) is a medical doctor and public health expert with a special interest in health counseling and individual health behavior. She started her academic career at a histo-pathology department where she examined skin diseases. Only Hanns Moshammer (HM), also a public health expert, has no close professional link to UV research. He enjoys outdoor activities and has experienced many sunburns and even snow-blindness in his life. This does not bode well for a good public health role model, but it helps to see the broader picture, unaffected by special knowledge. Our cooperation started when SS asked HM to cooperate in an Austrian project on UV radiation and health: She needed the project to sustain her monitoring network. She had the UV data, but she needed health expertise because this was part of the project call [5].

We soon realized that meteorologists and public health experts talk different languages. We also learned that health professionals have a somewhat simplified concept of UV radiation [6]. They know about two bands of the UV spectrum, i.e., UVA and UVB, and they know of some of the beneficial and adverse effects of UV exposure. However, to describe an optimal dose or dose rate that maximizes the beneficial and minimizes the adverse effects of the exposure, a deeper understanding of the complex interaction between the radiation and the exposed cells and tissues is mandatory. Therefore, we suggested dedicating the special issue to a very broad theme—“from physics to impact”—and not just to health impact alone. We are fortunate that the submitted papers cover a broad range of aspects including—among other things—exposure quantification [7,8] and UV as an agent for disinfection [9,10].

## 2. Reducing Melanoma Risk—The Issue of Health Literacy

Exceptionally high rates of melanoma skin cancer in the light-skinned descendants of British settlers in Australia set the stage for intensive public health debate about individual protection measures against UV radiation [11]. Destruction of the ozone layer and increasing ground level UV radiation also fueled this public health debate [12].

Lifetime risk for melanoma skin cancer is enlarged by exposure to natural and artificial UV light and reduced by sun protection. Thus, disease prevention is feasible simply and cost-effectively by appropriate photo-protection and sun avoidance. Increasing melanoma incidence and mortality rates worldwide create human suffering and a vast economic healthcare burden. To tackle this important global public health issue, costly skin cancer prevention programs have been initiated. These health campaigns strive at enhancing both knowledge and awareness regarding the potential dangers of UV radiation from natural and artificial sources. To supplement the clinical perception of skin disease diagnosis and treatment, we implemented the concept of public (skin) health as an umbrella term for conceptual and pragmatic research in the field of skin health promotion and cancer prevention.

Austrian melanoma incidence and mortality rates are constantly rising with higher rates in males. With Austria being a predominantly mountainous country, our ecological study found that melanoma incidence rates increased, whereas mortality rates decreased with altitude of place of living [5]. The observed diverging incidence and mortality trends might be explained by diagnosis at earlier tumor stages due to better screening adoption in these regions and/or vitamin D-driven slower tumor progression. Additionally, upwelling radiation caused by e.g., sunlight reflected by snow cover could also explain higher melanoma incidence rates with altitude [7].

We further aimed at investigating prevailing sun exposure and photo-protective practices influencing skin health among the general Austrian population. When exploring gender-specific aspects of recreational skin health habits, we identified a male factor, suggesting higher prevalence of sun exposure and poorer sun protective behavior among males [13,14,15].

Among the Austrian population, print media and television are perceived as the two most relevant sources for skin health information, thus ranking as sources before physicians [16,17,18]. Compared to various other information sources, respective information provided by doctors seems to positively influence knowledge on skin risk and sun-protective behavior. In respect to the known publishing source bias of information material, there is an urgent need for monitoring and standardizing the content of skin health educative information [18].

Despite media campaigns on the harmful effects of excessive sunlight and sunbed exposure, we found a high prevalence of self-reported sun exposure and sunbed use among Austrian citizens [19,20]. Social acceptance and norms influence tanning habits and sun-safe practices [21]. These findings highlight the demand for targeted health messages to attain lifestyle changes towards photo-protective habits. Providing resources that encourage pro-active counseling in everyday doctor–patient communication could increase skin health knowledge and sun-protective behavior, and thus curb the rise in skin cancer incidence rates. Communicating individualized public (skin) health messages might be the key to prevent photo-induced skin health hazards.

We are pleased that we were able to invite additional papers dealing with this aspect [22].

## 3. The Bright Side of the Sun—Beneficial UV Effects

From a public health point of view, the beneficial health effects of UV exposure are also of relevance. Additionally, the positive impact of physical outdoor activity in spite of and in conjunction with solar UV radiation cannot be neglected [15]. In the beginning, it was not so easy to encourage submission of papers that highlight this aspect. However, we are finally happy to also see some good work in our special issue dedicated to beneficial UV effects spanning from experimental [23] to epidemiological approaches [24,25,26]. The technical application of UV radiation for disinfectant purposes is another beneficial aspect [9,10].

## 4. Conclusions

Human perception, cultural norms, and health behavior are often strange and unpredictable. Tanning is now perceived by many as a cosmetic measure enhancing good appearance [21]. There are cultures where a light skin is deemed a sign of beauty. People even use whitening creams containing toxic ingredients such as mercury to enhance their beauty [27,28,29]. Others are so mortally afraid of the sun that they use protective creams that contain ecologically harmful substances such as endocrine disruptors [30,31] or nano-sized metal oxides [32,33], the ecosystem fate and effect of which have not been sufficiently studied. We know men who look down on their fellow citizen simply because he has dark skin but they themselves take every trouble to get their own skin tanned strongly and completely. Many more pay money to get a regular dose of UV radiation although this service is offered free of charge by nature. Public health experts must navigate between these conflicting beliefs and attitudes and give sound and reasonable advice. We do hope this special issue will help them with their task.
